# Prioritizing conservation investments for mammal species globally

**DOI:** 10.1098/rstb.2011.0108

**Published:** 2011-09-27

**Authors:** Kerrie A. Wilson, Megan C. Evans, Moreno Di Marco, David C. Green, Luigi Boitani, Hugh P. Possingham, Federica Chiozza, Carlo Rondinini

**Affiliations:** 1 School of Biological Sciences, The University of Queensland, St. Lucia, Queensland 4072, Australia; 2 Information Technology Services, The University of Queensland, St. Lucia, Queensland 4072, Australia; 3 Global Mammal Assessment programme, Department of Biology and Biotechnologies, Sapienza Università di Roma, Viale dell'Università 32, 00185 Rome, Italy

**Keywords:** spatial conservation prioritization, mammals, threat abatement, threatened species, likelihood of success, protected areas

## Abstract

We need to set priorities for conservation because we cannot do everything, everywhere, at the same time. We determined priority areas for investment in threat abatement actions, in both a cost-effective and spatially and temporally explicit way, for the threatened mammals of the world. Our analysis presents the first fine-resolution prioritization analysis for mammals at a global scale that accounts for the risk of habitat loss, the actions required to abate this risk, the costs of these actions and the likelihood of investment success. We evaluated the likelihood of success of investments using information on the past frequency and duration of legislative effectiveness at a country scale. The establishment of new protected areas was the action receiving the greatest investment, while restoration was never chosen. The resolution of the analysis and the incorporation of likelihood of success made little difference to this result, but affected the spatial location of these investments.

## 1. Introduction

Conservation expenditure must be prioritized so that scarce funds and resources are used efficiently and effectively to prevent long-term loss and degradation of natural systems. Various templates for prioritizing the investment of conservation funds at a global scale have been developed, each with different objectives [1]. Past prioritization analyses for mammal conservation have identified the minimum area required to achieve pre-specified targets and have typically been conducted at a coarse resolution (e.g. 10 000 km^2^) [2,3]. Other global-scale analyses for mammals have delivered important information on the coverage of the global protected areas (PAs) network [4,5], on the drivers of extinction and biogeographic patterns [3,6–9] and on the surrogacy value of mammals for other taxonomic groups [10–12]. A fine-resolution prioritization analysis at a global scale for any taxonomic group, including mammals, has been precluded to date owing to the lack of fine-resolution species distribution data [1]. The availability of such data for mammals from the recently completed Global Mammal Assessment (GMA) [13] presents an important opportunity to address this gap and improve decisions about where funds should be expended to conserve biodiversity. However, a comprehensive prioritization analysis at a global scale must also account for: the vulnerability of sites, the actions required to abate threatening processes, the costs of these actions and their likelihood of success [14,15].

Typically, the implementation of conservation activities is a protracted process and the investment landscape will change through time as areas are protected or destroyed. The cost-effectiveness of investments can be enhanced and the likely persistence of species can be improved by explicitly accounting for landscape dynamics [16–18]. While several of the global prioritization templates incorporate a measure of vulnerability [1], the measures employed are static, generic measures and therefore cannot inform when or how funds should be spent. Until recently, there has been a deficit of spatially explicit data on the rate of habitat loss owing to a range of threatening processes, which is essential to inform the relative priority for investing in different actions in different locations through time [2,19,20]. There are two areas of dynamic conservation planning that are largely unresolved in the literature. First, the exact solution to a dynamic conservation planning problem requires the application of stochastic dynamic programming [18,21,22] in order to find optimal solutions. As large conservation problems are impossible to solve using this method, we have the technical challenge of deriving robust approximations. Second, there is some confusion about how the process of protection influences threats. In some analyses, threatened sites are avoided, presumably because protection will not stop the threat [23]. In other analyses, threatened sites are preferred because protection will abate the threat. In reality, many situations lie between these extremes—protection may partially mitigate the threat, or mitigate the threat with an uncertain probability [24–26].

It is impossible to provide direction on how funds should be allocated without considering the actions required to abate specific threats. Habitat loss and degradation are the key threats to mammal persistence [9,27]. Globally 5000 million hectares of native vegetation have been converted to agriculture [28] and there are extensive areas which have been cleared for agriculture that now require restoration [29]. While designating PAs might stop or slow the conversion of habitat, there is often also a need to reverse habitat degradation to ameliorate the effects of past land use decisions [30,31]. Similarly, many land-management strategies require adjustment to enhance their suitability for biodiversity [32]. For example, reduced impact logging (RIL) practices have been identified as a priority action to conserve biodiversity in tropical forest regions [33,34]. Recent advances in conservation planning facilitate the prioritization of multiple conservation actions, ranging from protection to restoration [35,36]. Such multiple-action analyses have to date been restricted to local or regional scale situations.

None of the existing global prioritization templates incorporate costs directly [1], let alone account for the spatially variable costs of different conservation actions. There is now sufficient evidence of the importance of explicitly incorporating economic data into prioritization analyses, with conservation plans that are up to 10 times more efficient [37] and conflicts with areas important for agriculture halved [2]. The identification of priority areas has also been found to be more sensitive to the inclusion of cost data as opposed to biodiversity data, emphasizing the need to consider both ecological and economic data in prioritization analyses [38].

Funding commitments are likely to require adjustment through time with changes in political and institutional capacity and stability. Between 1950 and 2000, over 90 per cent of the major armed conflicts in the world occurred within countries containing biodiversity hotspots, and 80 per cent took place directly in biodiversity hotspot areas [39]. In general, the links between governance and the loss of biodiversity are complex [40–42] but conflicts have been found to result in habitat destruction as a result of an ineffective enforcement [40,43,44]. This means that the chance of successful investments varies from place to place but this variation has not been incorporated in global prioritization templates [1,42,45]. With biodiversity hotspots concentrated in politically volatile regions, it has been recommended that the conservation community maintains continuous engagement during periods of conflict [39]. However, concerns regarding security and instable governance can force the suspension of conservation activities [46,47], and international aid can shift to peacekeeping and humanitarian efforts [40]. Eklund *et al*. [48] find that when poor governance (specifically, corruption) results in much higher management costs, conservation budgets are more effectively allocated to countries with better governance. If we assume that periods of ineffective legislature will reduce the funding available for conservation then the probability (and likely duration) of such interruptions can be explicitly accounted for when prioritizing conservation spending [45].

In this paper, we provide a logical and comprehensive prioritization framework for conservation expenditure at a global scale [14]. The objective of our analysis is to maximize the number of species conserved, through strategic investment in a suite of conservation actions, given a fixed budget constraint. In doing so, we account for the following information:

— mammal species distribution data;
— the main threats to each species and the probability of habitat conversion owing to each threat;
— the actions likely to abate the threats and the costs associated with the implementation of these actions; and
— the likelihood of investment success.

Our aim is to determine the amount of funds to be directed towards each conservation action in a spatially and temporally explicit manner. Our analysis therefore represents the first comprehensive, multiple-action, prioritization analysis undertaken at a fine resolution at a global scale.

## 2. Methods

### (a) Spatial data

We used an equal-area grid to delineate sites available for conservation investment and conducted our analysis at two spatial scales: 10 × 10 km and 30 × 30 km. There are 1 141 064 sites at a 10 km resolution occupied by the mammal species of concern in this analysis (see below) and approximately 135 470 sites at a 30 km resolution. Grid layers were clipped to the spatial extent of the 186 countries included in the analysis (the countries for which data used in the analysis were available) meaning that sites located along the coastline are of irregular shape.

We classified land within each site into five primary land uses using the GlobCover global land cover map and the 2010 World Database on Protected Areas (for further information see electronic supplementary material, appendix 1). For each grid cell, we calculated the area (in square-kilometres) contained within PAs [49], excluded areas [50], agricultural land [50], non-intact closed native forest likely to be subject to human disturbance [51] and unallocated land [50]. We considered unallocated land to be areas of native vegetation that may be subject to habitat conversion and available for the designation of PAs.

We used fine-scale habitat suitability models for terrestrial mammals [13] to calculate the total area of suitable habitat (in square-kilometres) for each species contained within each site. We considered Threatened and Near-Threatened species, which together represent 21 per cent of terrestrial non-extinct mammals (*n* = 1128). We also accounted for Data-Deficient species (*n* = 779; 15 per cent of terrestrial non-extinct mammals). We then extracted information on the threats to each species from the IUCN Red List database [52]. We obtained information on the threats affecting the status of 1669 species (178 Critically Endangered species, 437 Endangered species, 473 Vulnerable species, 292 Near-Threatened species and 289 Data-Deficient species). Of these species, 1372 are threatened by agriculture/farming and/or forestry activities, with 430 species affected only by agriculture/farming, 195 species affected only by forestry activities and 747 species affected by both threats. We therefore considered two threatening processes: the conversion of natural habitat to (i) agriculture and (ii) plantation forestry. Threat information contained within the GMA database identifies species that are currently threatened, and therefore only reflects responses to past land transformations. Our analysis does not consider species which may potentially be affected by a threat once land use change occurs [53].

### (b) System dynamics

We considered the future vulnerability of each site to land use conversion by agriculture and plantation forestry. We determined the rate of conversion to agriculture using the GLOBIO3 dataset and drivers of land use change from the Integrated Model to Assess the Global Environment [19]. Predictions were generated over 20 years (from 2010 to 2030) using the scenario ‘order from strength’, which represents the worst-case scenario in terms of habitat loss owing to agricultural development [54]. We determined the annual rate of conversion to plantation forest by applying the past rates of forest conversion (from 2000 to 2005) to the area of intact forest within each site [55]. We determined the percentage of the total forest area in each country that contained plantations in 2005 [56] and using the annual rate of conversion estimated the proportion of the remaining forested area that might be converted to plantations in the future. We did not consider the possibility of contagion in habitat clearance between sites, or feedbacks in the rate of habitat conversion in a site over time; hence the annual rate of land conversion to agriculture and plantation within each site remained constant over our analysis time period, but the area converted in each site reduced through time as land was protected or converted.

### (c) Costs of conservation action

We considered three conservation actions for investment: the establishment and management of new PAs within unallocated land, restoration of land cleared for agriculture and RIL. The costs of implementing a conservation action are a composite of the cost of land purchase, management, foregone returns and transaction costs [57]. We determined the cost of new PAs in our analysis ($US per hectare per year) as the sum of the opportunity cost of forgone agricultural rents [58] and the predicted cost of PA management [59,60]. We endowed this summed annual cost using a discount rate of 9 per cent to determine the total cost of protected area implementation within each site ($US per hectare) over the entirety of our 20-year planning period (for further information see electronic supplementary material, appendices 2 and 4).

RIL was restricted to non-intact forest in tropical developing countries. We determined countries that were considered to be still developing [61], and from those selected developing countries where more than 50 per cent of land area was contained within the tropics. It has previously been found that RIL does not incur an opportunity cost as it can yield more timber and incur lower harvesting costs than standard logging practices [62,63]. We determined the cost of RIL using data on the costs of training personnel in RIL techniques. We accounted for the cost of training concession operators every 5 years in RIL practices (estimated to equate to US$11 per hectare [64]), and we endowed this cost over 20 years (using a discount rate of 9%), which equates to US$50 per hectare and assigned this as a flat cost to each site where RIL was an available action for conservation.

We assumed that restoration may only occur on agricultural land in sites where there is a zero rate of habitat conversion, as restoration is unlikely in a site still being cleared for agriculture. Restoration on agricultural lands will bear an agricultural opportunity cost, and also an implementation cost. The implementation cost of restoration varies with site-specific characteristics that are impossible to account for at a global scale. We used a standard cost of restoration in tropical forest areas of US$12 150 ha^−1^ [65] for all locations and assigned this cost (combined with the endowed cost of foregone agricultural rent) to each site where land was available for restoration.

### (d) Likelihood of conservation investment success

We quantified the likelihood of investment success for each site using data obtained from a time-series database on legislature effectiveness [66]. Annual probabilities of investment failure were determined according to the number of incidences of complete legislature ineffectiveness in each country between 1980 and 2005: for one incidence during this timeframe, a probability of 4 per cent of investment failure was assigned to the country (=1/24, applicable to 39 countries); two incidences resulted in a probability of 8 per cent (applicable to 11 countries) and three incidences equated to a 12.5 per cent probability of investment failure (applicable to two countries). We considered the possibility that funding could resume in countries where the ability to invest had previously been lost, and calculated the probability of funding resuming was approximated by the inverse of the average number of years a country has had an ineffective legislature from 1980 to 2005. We determined whether funding was possible or not in a particular year by comparing the probability of investment failure at each site to a random number drawn from a uniform distribution of interval (0, 1).The algorithm was run 100 times and the investment allocations were averaged.

### (e) The conservation prioritization approach

The objective of our analysis was to maximize the gain in species protection, given a fixed budget for conservation investment (a detailed explanation of the mathematical framework is provided in electronic supplementary material, appendix 3). We employed the current annual budget from the Global Environmental Facility, which equates to $968 million (see http://gefeo.org/interior_right.aspx?id=48). Our management decision was to determine which conservation action should be undertaken where and when, by maximizing the expected utility *E*[*Y*_*ij*_] gained from investments into conservation actions:
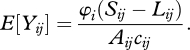
The benefit gained from investing in action *j* is equal to the sum of suitable habitat for all species (relative to the total area of suitable habitat for each species globally) within site *i* which would be protected by that action (*S*_*ij*_), minus any expected loss of habitat that may occur in that site (*L*_*ij*_) as during the following timestep (see electronic supplementary material, appendix 3). The total cost investing in an action within a timestep is the product of the amount of area within a site available to implement a particular action (*A*_*ij*_), and the cost per unit area of that action (*c*_*ij*_). Therefore, the expected utility of a site-action combination in a particular timestep is the quotient of the overall benefits and cost, multiplied by the likelihood of investment success in a site (*φ*_*i*_).

Species threatened by both threatening processes were simultaneously assumed to only be adequately protected through the establishment and management of PAs, whereas species affected by only one threat may benefit from PAs and one of the other two actions. Of our three potential conservation actions, only the establishment and management of PAs prevented habitat conversion, and only one action was able to be implemented at each site during each timestep. The establishment and management of PAs were therefore favoured in sites where the habitat lost was greater than the species habitat that could be gained through restoration or RIL. There was a time lag of 15 years before the benefits of restoration were realized.

We assumed a linear accumulation of benefit to a species with respect to the allocation of investment towards conservation actions over time. To avoid bias in the prioritization analysis towards species with large ranges, we devised species' protection targets scaled according to their global area of occupancy. Once species had achieved their pre-specified targets, they were removed from the analysis such that their presence did not further influence the funding allocation process, and no further benefit was accumulated. We set the target thresholds at 100 per cent of the distribution of each species with an area of occupancy of less than 500 km^2^ (22.7% of species), 20 per cent of the distribution of each species with an area of occupancy of greater than 125 000 km^2^ (18.5% of species) and 20–100% of the distribution of each species with an intermediate area of occupancy (interpolated using a log transformation) (58.8% of species) [4].

Finding an optimal solution to such a large problem is computationally intractable, so we employed a myopic greedy investment algorithm to identify a near-optimal investment schedule over a 20 year time period [18,67] (see electronic supplementary material, appendix 3). Continuous portions of area within the 10 or 30 km grid cells were selected based upon the amount of money available and the area available for an action.

We compared our analysis with a scenario where the likelihood of investment success in the selection of sites for investment was ignored. In this case, the algorithm did not respond to variation in the likelihood of investment success, but if funds were allocated to countries where the legislature was considered to have failed, then the investment did not contribute towards the achievement of the species' targets. We also conducted our analysis at both a 10 and a 30 km resolution. Electronic supplementary material, appendix 4 contains a summary of the input data employed.

## 3. Results

At a 10 km resolution, approximately 9 million km^2^ was selected for investment and the average cost of the sites selected for investment was US$351 776, with the average area being selected in a site during one timestep being 60 km^2^. Nine species had no contribution towards their target. There was very little difference between the 10 and 30 km resolution datasets in terms of the budget and area allocated to each action globally. Using either dataset, 93.6 and 6.4 per cent of the budget and approximately 97.5 and 2.5 per cent of the area were allocated to PAs and to RIL, respectively. Restoration was not allocated any funding using either dataset. While priority countries remain the same, the budget allocated to the top countries varies with resolution. There were however differences in the total allocation to each country and the rank ordering of countries in terms of their total recommended investment (table 1 and see electronic supplementary material, appendix 5). Species also exceeded their target by a greater extent (prior to being removed from the analysis) using the 30 km grid cell dataset and therefore fewer species met their targets after 20 years (338 species versus 378 species met their targets, respectively, using the 30 km versus the 10 km grid cell datasets). The greatest extent to which a target was exceeded was 2 per cent at the 10 km resolution and 7 per cent at the 30 km resolution.

**Table 1.** The funding and area allocated to (a) protected areas (PAs) and (b) reduced impact logging (RIL) for the top five countries receiving investment at a 10 km resolution (the results for all countries are provided in electronic supplementary material, appendix 5). Values represent average allocations over 20 years from 100 runs of the investment allocation algorithm.

(a) country	US$ allocated to PA after 20 years (millions)		area allocated to PA after 20 years (km^2^)	
	10 km	30 km	10 km	30 km
Indonesia	1831	1979	195 045	188 606
Madagascar	1599	2116	69 109	88 641
Peru	1348	1255	57 386	55 217
Mexico	1319	1871	23 910	32 843
Australia	852	445	13 666	7 760
	US$ allocated to RIL after 20 years (millions)		area allocated to RIL after 20 years (km^2^)	
(b) country	10 km	30 km	10 km	30 km
Indonesia	216	222	43 261	44 586
Brazil	133	98	26 789	19 736
Colombia	103	88	20 694	17 738
Ecuador	84	100	16 897	20 145
Papua New Guinea	76	63	15 378	12 727

**Figure 1.**
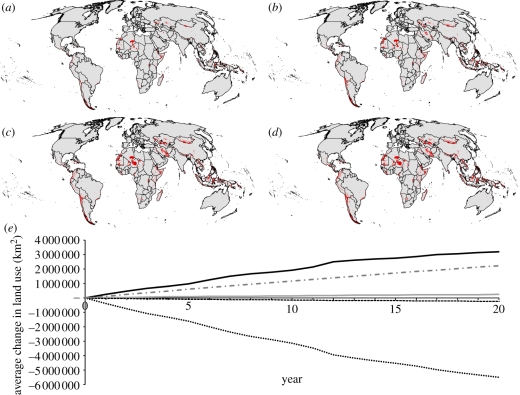
Spatial distribution of conservation funds through time at (*a*) 5, (*b*) 10, (*c*) 15 and (*d*) 20 years for all conservation actions, and (*e*) the average change in land use through time. Restoration received no investment after 20 years. Black solid line, protected areas; grey line, reduced impact logging; black dashed line, forestry; black dotted line, unallocated; grey dashed-dotted line, agriculture.

**Figure 2.**
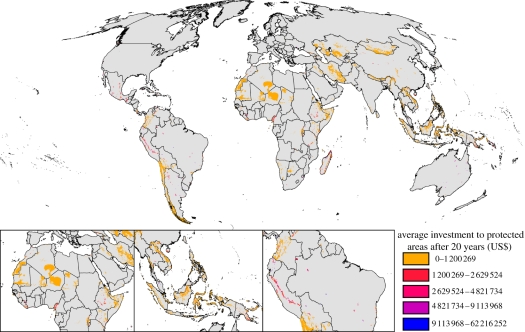
Average total investment (US$) in protected areas after 20 years.

**Figure 3.**
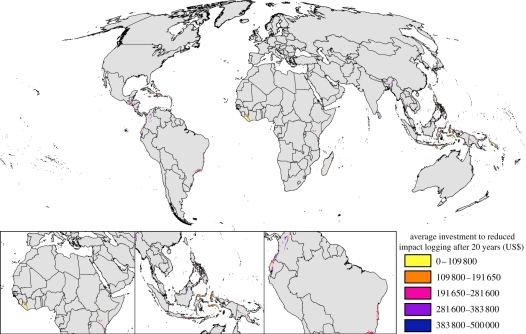
Average total investment (US$) in reduced impact logging after 20 years.

Through time, the area of unallocated land is reduced as land is either protected, converted to agriculture or assigned to RIL (figure 1). We find that approximately 30 per cent of the total funding allocated in the first 5 years was directed towards Madagascar and Indonesia. Overall, 91 countries received funding in the first 5 years, 101 countries in the first 10 years and 112 countries after 20 years. PAs received the greatest proportion of the total budget over 20 years (just over US$18.1 billion) with RIL receiving just over US$1.2 billion. Indonesia received the greatest amount of funding allocated to PAs over the 20 years, with Madagascar, Peru, Mexico and Australia also receiving high levels of investment (table 1(*a*), figure 2 and electronic supplementary material, appendix 5). The greatest investments in RIL were also directed towards Indonesia, with Brazil, Colombia, Ecuador and Papua New Guinea also favoured for investment (table 1(*b*), figure 3 and electronic supplementary material, appendix 5).

**Figure 4.**
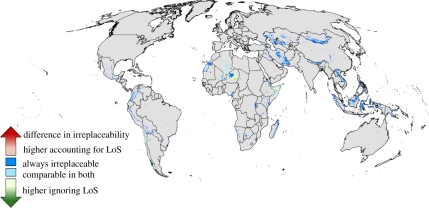
Comparative irreplaceability of sites (the frequency of selection of each site in 100 runs of the investment algorithm) when the likelihood of investment success (LoS) is explicitly accounted for in the selection of sites and when it is ignored. ‘Comparable in both’ refers to sites where the difference in irreplaceability is less than 5% regardless of whether the likelihood of success is accounted for. Sites that are always irreplaceable have a selection frequency of 100%.

Accounting for the likelihood of success in the allocation of funds did not influence the average area and budget allocated to each action, but rather the locations receiving investment differed (figure 4). For example, some countries received greater investment when the likelihood of investment failure was not accounted for (e.g. Ethiopia, Liberia, Myanmar and Nigeria). Seven fewer species met their targets when the likelihood of investment success was not explicitly accounted for but the level of target achievement for these species was still greater than 95 per cent.

## 4. Discussion

We have developed and applied at a global scale a comprehensively formulated approach for prioritizing investments in the protection of biodiversity. While we focus on mammal species in this analysis, we do not advocate their use as surrogates for other species or biodiversity as a whole [10,68]. Our approach is not limited to any particular taxonomic group and fine-resolution data for additional taxonomic groups could be readily incorporated into our analysis as it comes available. It is however likely that the socio-economic factors that we have included here will be more important determinants of investment priorities than the choice of biodiversity data [38].

Our prioritization analysis has employed fine-resolution species distribution data from the GMA providing greater accuracy in the spatial distribution of biodiversity features than was available previously. But the downside of this level of detail is that the allocation of funds is computationally exhaustive and time-consuming at a global scale for even specialized, high-performance computers. Using ecoregions, or some other broad geographically defined unit of analysis, would produce results far more quickly, but would fail to adequately capture the spatial heterogeneity that exists in the distribution of threats, species and costs. Even for our analysis at a 10 and 30 km resolution, we assumed a random distribution of species and land uses within each site. By comparing the results from a 10 km grid cell dataset with a 30 km dataset, we find a reduction in the number of species that achieve their targets over 20 years and that the total amount of funding recommended to be allocated to each country differed depending on the resolution of dataset employed. With increasing computational capacity and the availability of fine-resolution data, it is clear that fine-resolution data should be employed wherever possible and analyses should be undertaken at an appropriate scale to inform implementation [15]. Our analysis does however illustrate that it is now possible to undertake a fine-resolution, bottom-up prioritization analysis at a global scale, where the analysis is driven by the conservation actions required to abate the main threats to the species of interest [36].

The priority areas consistently identified by existing global prioritization templates are in the tropics and Mediterranean environments [1]. We also found that such regions were favoured for investment using our prioritization approach, with the additional preference given to the temperate regions of South America and the arid and semi-arid regions of Central Asia. In general, conservation attention has to date been directed to the threatened species inhabiting the Australasian and Nearctic regions, with tropical and Palearctic regions receiving the least attention [69]. Current conservation efforts in Indonesia are considered underfunded compared with the relative priority of this country for mammal conservation, and the same applies also to other countries identified as priorities in our analysis such as Madagascar, Mexico, Columbia, Brazil and Papua New Guinea [2]. We find that Indonesia is the highest priority country for investment, for both RIL and the establishment and management of PAs. This also reflects the results of previous global prioritization analyses undertaken at a coarser resolution [2], but we add value to these previous analyses by accounting for landscape dynamics in the allocation of funds [18] and thereby provide a temporal allocation of funding identifying not only where, but also how and when funds should be allocated to conserve mammals globally.

PAs are favoured in our analysis, although a small proportion of funding is also directed towards RIL in tropical countries with significant forested areas that are subject to human disturbances, such as Indonesia, Columbia, Brazil and Papua New Guinea. Restoration was not prioritized within the time frame of 20 years that we evaluated and as a consequence some species would never be able to achieve their targets [70]. Overall, the number of species that could benefit from RIL and restoration and the level of variation in this number was less than that for the action of protecting areas. Restoration was further disfavoured as this action was not permitted to occur in any site that is still losing native habitat (there were 148 257 sites available for restoration compared with 1 141 025 sites available for protection at the 10 km scale). The nature of our objective function, to maximize gains given a fixed budget, also disfavoured expensive actions such as habitat restoration. Previous studies have shown the cost of conservation to drive the selection of sites for investment [38,71], and this effect is emphasized where the variation in cost is much greater than the variation in benefits (in our study the costs of protection varied by approximately six orders of magnitude, whereas the benefits varied by only two).

We assumed that all threatened species would benefit from PAs and that species impacted simultaneously by forestry and agriculture would only persist if both threats were mitigated, hence the establishment and management of PAs was the only action that was made available to protect these species. To resolve the choice of action in such cases would require determining the probability of occurrence of each threat in each site. This could be achieved if more accurate rates of conversion data were available at a fine resolution, which could be obtained via evaluating the economic value of each parcel of land under agriculture versus plantation forestry [72].

We included a 15-year time lag associated with when the benefit of restoration was realized; however this time lag was not explicitly considered during the prioritization process and hence would not have influenced whether the action was selected or not. The incorporation of restoration time lags in our analysis could be further improved by adapting the algorithm to account for the presence of the time lag when selecting site/action combinations for investment [73,74]. This is likely to disfavour restoration even further, but would reflect a more accurate problem formulation.

Greater variability in the costs of RIL and restoration would have probably seen these actions selected more frequently. Accounting for the spatial heterogeneity of the costs of restoration and RIL and of the spatial distribution of the threatening processes we have evaluated would therefore refine our assessment of priority areas for conservation investment. Similarly, refined data on conservation opportunities and constraints for each conservation action would improve the analysis we have presented [75]. While restoration was not favoured in our analysis, restoration would probably be selected in the analyses undertaken at a smaller spatial scale, in countries with little ongoing habitat loss and where the ratio of the cost of restoration compared with the cost of PAs was much closer to one.

Our choice of objective function accounted for the expected consequences of choosing one action over another within a particular site. The benefit gained from investing in an action was countered by its ability to prevent further loss of species habitat within the next timestep. However, the current objective function does not differentiate between sites in terms of their relative vulnerability. That is, while we were able to select the action that achieved the greatest expected gain within a site, sites with the greatest risk of habitat loss were not necessarily chosen for investment [20]. The choice of either maximizing gains or minimizing losses depends upon whether a proactive or reactive approach to conservation investments is desired [14,76]. Minimizing losses will also be more appropriate when the levels of threat to each site differ greatly and under such circumstances a more risk-averse approach to investment allocation will deliver better outcomes [18,77]. However, the choice of approach is complicated in the context of multiple conservation actions as some actions abate future habitat loss and degradation and others ameliorate the impacts of past pressures. The development of investment algorithms that combine two objective functions, minimizing loss and maximizing gains, within a multiple action framework represents an important area of future research.

We recorded the progress made towards meeting the target for each species as funds were allocated through time, however our framework did not explicitly consider the contribution towards each target when calculating the benefit of investing in an action in a particular timestep. An alternative formulation of our framework may be to scale the benefit of investments according to the extent that targets for each species are currently achieved as it may be preferable to invest in an action to protect species that have made little progress in meeting conservation targets compared with species that were close to meeting their target.

An important data limitation for undertaking global scale prioritization analyses is knowledge of the current distribution of plantation forests, the risk of conversion of native habitat to plantations, and the predicted future extent of plantation forests. Most data on plantations is at country level and is non-spatial. The dataset on the rate of conversion to plantation that we created assumes that past conversion rates to plantations represent future rates. We also assumed that all non-intact areas of forest are subject to human disturbances, which is probably an overestimate of this area. The development of fine-resolution, spatially explicit maps of current plantations is a key data gap that requires addressing in order to facilitate future prioritization analyses that account for multiple conservation actions. Similarly, direct killing of mammals (e.g. through bushmeat hunting or poaching) is a significant threat to mammal persistence [78] but was not considered in our analysis owing to a paucity of data at a global scale on the spatial distribution of this threat. If such data were available then a simplistic way of accounting for the costs associated with abating this threat would be to factor in the costs of mitigation actions (e.g. park patrols, compensation or public education programmes) into the costing of RIL and PA management. An area of future investigation is to evaluate the relationships between investments in this action and the likelihood of investment success associated with political instability [79] and habitat loss [80,81].

Our analysis has facilitated an assessment of the impact of a conservation organization continuing to invest in a country even though the legislature is considered ineffective, based on the assumption that future investments will not succeed in achieving their conservation goals as funds might be misused (or directed towards other important needs). We find that the achievement of targets is only marginally reduced if the likelihood of investment success is ignored. There are probably two reasons for this. First, there were numerous site-action combinations available for investment and therefore funds were able to be diverted towards sites with a greater likelihood of success without impacting the overall result. Second, when we ‘ignored’ the likelihood of success, funds were able to be invested in countries where the legislature is considered ineffective, but no benefit was realized from this investment. However, because of the generally low cost of these sites and the comparatively similar average species richness for each site in countries with a higher likelihood of success, the impact on the overall results was minimal. Our approach to dealing with the probability of investment success is quite conservative: the costs of conservation remained stable [48] and sites that had already been selected for investment were not affected by the cessation of funding as only future investments were impacted. Future analyses will explore the impacts of ignoring the likelihood of success of all investments: past, present and future.

We have developed and applied the first comprehensive and dynamic approach to prioritizing the allocation of conservation spending at a global scale that considers the costs, benefits and likelihood of success of a range of conservation actions that will abate the main threats to species persistence. We estimate that the budget required to protect 10 per cent of the remaining native habitat is approximately US$32 trillion, significantly greater than the budget of approximately US$20 billion accounted for in this analysis. Such gaps further emphasize the value of prioritization approaches, such as the one we have developed and applied, and that can assist with understanding what investments need to occur where and when, to deliver the greatest return.
